# Comparative evaluation of micronutrient status in the serum of diabetes mellitus patients and healthy individuals with periodontitis

**DOI:** 10.4103/0972-124X.65439

**Published:** 2010

**Authors:** Biju Thomas, Suchetha Kumari, K. Ramitha, M. B. Ashwini Kumari

**Affiliations:** *A.B. Shetty Memorial Institute of Dental Sciences, Mangalore – 575 018, India*

**Keywords:** Diabetes mellitus, micro nutrients, nutrition, periodontitis

## Abstract

**Background:**

Periodontal diseases are microbial induced chronic inflammatory conditions characterized by infiltration of leukocytes, loss of connective tissue, alveolar bone resorption, and formation of periodontal pockets. In response to periodontal pathogens, the leukocytes (PMN) elaborate destructive oxidants, proteinases and other factors. The balance between these factors, the antioxidants and endogenously synthesized antiproteinases determine the extent of periodontal damage. Diabetes mellitus is a metabolic disorder. Most of the complications of diabetes are due to hyperglycemia. Persons with diabetes are at a greater risk for periodontal disease Malnutrition is characterized by marked tissue depletion of antioxidant nutrients and impaired acute phase protein response to infections resulting in impaired healing. Diabetes mellitus also alters the micronutrient levels. Malnutrition is characterized by marked tissue depletion of antioxidant nutrients and impaired acute phase protein response to infections resulting in impaired healing. Malnutrition, which usually involves concomitant deficiencies of several essential macro and micro nutrients, therefore, has the potential to adversely influence the prognosis of periodontal infections. Objectives:This study has been conducted to evaluate and compare the serum levels of vitamin C, zinc and copper in diabetic and healthy individuals with periodontitis.

**Materials and Methods:**

In this case control study 60 subjects inclusive of both sexes were selected and divided into 3 groups of 20 each. Group 1 comprised of 20 subjects with type 2 diabetes mellitus and periodontal disease, Group 2 comprised of 20 healthy subjects with periodontal disease. And Group 3 comprised of 20 healthy subjects without periodontal disease. Venous blood samples were collected and centrifuged at 3000rpm for 15 minutes and the superanatant serum is collected to measure the vitamin C, zinc and copper levels. The vitamin C levels of clinical samples were measured using spectrophotometric quantitation (dinitrophenyl hydrazine method) and zinc and copper levels were measured using atomic absorption spectrophotometry.

**Results:**

The results showed that the levels of vitamin C and zinc decreased and copper levels increased in diabetic patients with periodontits compared to healthy individuals with periodontitis.

**Conclusion:**

It may be reasonable to suggest vitamin and/or mineral supplements for patients whose nutrition might be inadequate. Future research should focus on an evaluation of which nutrients may help to prevent the onset and progression of periodontal disease

## INTRODUCTION

Periodontitis is a term used to describe an inflammatory process, initiated by the plaque biofilm that leads to loss of periodontal attachment to the root surface and adjacent alveolar bone and which ultimately results in tooth loss. The primary etiological agent is specific, predominantly gram negative anaerobic or facultative bacteria within the subgingival biofilm. These bacteria have the ability to activate host defense mechanisms, which breakdown epithelia and other structures of gingiva and periodontium, while at the same time inactivating repair systems.

Although now it has been unanimously accepted that periodontal disease is the resultant of an interaction between microbial plaque and the resultant inflammatory and immunological changes within the periodontal tissues, it is also recognized that the nature and severity of this interaction in turn may be modified by many systemic factors, including hormonal changes, nutritional deficiencies, blood dyscrasias, drug ingestion, aging or a compromised immune system.

The vitality of the periodontal tissues, in both health and disease, depends strongly upon an adequate source of essential nutrients being available to the host. The epithelium of the dento-gingival junction and the connective tissue are among the most dynamic tissues in the body. The maintenance of these tissues and, therefore, the integrity of periodontium is dependent upon adequate supply of proteins, carbohydrates, fats, vitamins and mineral salts. A chronic deficiency in the availability of one or more of these nutrients may be expected to produce pathological alterations in the periodontal tissues.

The food we eat contains nutrients. Nutrients can be considered major or minor, as determined by the amounts consumed in our diet. Major nutrients are consumed in gram quantities and they include protein, carbohydrates, lipids. Micro nutrients are required in milligram to microgram quantities and include vitamins and minerals.

Periodontal diseases are the result of bacterial infections to the gingival tissues. Therapy to decrease the levels of oral microorganisms can reduce gingivitis and stabilize periodontitis. Although dietary components play a major role in the pathogenensis of dental caries, diet plays primarily a modifying role in the progression of periodontal disease. A periodontal lesion is essentially a wound, and sufficient host resources must be available for optimal healing to take place.

The exact mechanism by which nutritional deficiencies modify periodontal destruction has not yet been precisely defined. The effect of nutrition on the immune system and its role in periodontal disease has been recently reviewed. Nevia *et al*. in 2003 reviewed the literature on the use of specific nutrients to prevent and/or treat periodontal diseases and concluded that although treatment of periodontal disease with nutritional supplementation has minimal side effects, the data on its efficacy are limited. Diabetes mellitus is a group of complex multisystem metabolic disorders characterized by a relative or absolute insufficiency of insulin secretion and or concomitant resistance to the metabolic action of insulin on target tissues.

Diabetes mellitus is a major health problem in the world. Approximately 5% of the diabetics are classified as Type- 1(IDDM), a condition characterized by abrupt onset at any age, destruction of pancreatic islet cells, and dependence on exogenous insulin. The more prevalent form of diabetes is Type- 2 (NIDDM), a condition which often develops over a period of time, involves reduced responsiveness of tissues to circulating insulin, and is often controlled by diet or oral hypoglycemic drugs. Both types are characterized by hyperglycemia, hyperlipidemia, and associated complications.

Hyperglycemia is a hallmark of diabetes mellitus as are its chronic metabolic complications. Periodontitis has been recognized as the sixth major complication of diabetes

Among the systemic factors, the relationship between periodontal disease and diabetes mellitus has been studied extensively. Many investigators, in their epidemiological, experimental and clinical studies have reported that the severity of periodontal diseases is significantly greater among diabetics than in non- diabetics. Malnutrition has been suggested as a cause of diabetes mellitus. Numerous studies have found alterations in micronutrient status of patients with diabetes mellitus, and in some studies deficiency of certain minerals or vitamins has been correlated with presence of diabetic complications. However, the exact pathogenetic role of malnutrition in diabetes mellitus has been disputed.

Hence this study has been designed to estimate and compare the micronutrient levels of vitamin C, zinc and copper in diabetic patients and healthy individuals with periodontitis.

### Review of literature

A study to compare the serum levels of copper, zinc and magnesium in diabetic patients concluded that diabetes can alter copper and zinc levels. Perturbations in mineral metabolism are more pronounced in diabetic populations. But it is not clear whether differences in trace element status are a consequence of diabetes or alternatively, whether they contribute to the expression of disease.[1]

A case control study was conducted to evaluate the serum metal status in patients with noninsulin diabetes mellitus. They concluded that diabetics had lower zinc levels and higher copper levels than controls. There is also evidence of significant difference in metal status between diabetic patients with or without specific complications. This further indicated that patients with noninsulin diabetes mellitus have distinct changes in their metal status, and these perturbations are associated with some diabetic complications.[2]

A study was conducted to evaluate the effect of dietary intake of vitamin C and the presence of periodontal disease. Dietary intake showed a statistically significant relationship between with periodontal disease status, as measured by clinical attachment loss. Those taking the lowest levels of vitamin C had the greatest negative clinical effect on their periodontal tissues.[3]

Lack of vitamin C has been related to an increased risk of periodontal disease in a clinical study. The serum levels of vitamin C were significantly lower in peridontitis patients compared to healthy controls; consumption of grape juice for two weeks led to an increase in plasma vitamin c levels and reduced gingival sulcus bleeding scores.[4]

A study was conducted to evaluate the serum zinc levels and severity of periodontal disease. It concluded that patients with decreased zinc levels had increased alveolar bone resorption.[5]

A study was conducted to evaluate serum copper concentrations in diabetic individuals and healthy individuals. The results concluded that serum copper levels are higher in diabetic patients than healthy individuals.[6]

### Aims and objectives

1. To estimate the level of vitamin c, zinc and copper in serum of diabetes mellitus patients with periodontitis
2. To estimate the level of vitamin c, zinc and copper in serum of healthy individuals with and without periodontitis
3. To compare the level of vitamin c, zinc and copper in serum of Type 2 diabetes mellitus patients and healthy individuals with periodontitis

## MATERIALS AND METHODS

### Source of data

The subjects for this study were selected from the outpatients, Department of Periodontics, A.B. Shetty Memorial Institute of Dental Sciences, Deralakatte, Mangalore

### Method of collection of data

The study was designed as a case–control study comprising of 60 subjects, inclusive of both sexes and were divided into three groups of 20 patients each:

Group I: 20 patients of Type 2 Diabetes Mellitus with periodontal disease

Group II: 20 healthy subjects with periodontal disease

Group III: 20 healthy individuals without periodontal disease

### Criteria for selection

#### Inclusion criteria

1. Patients categorized as Type 2 diabetes mellitus should have
  - random blood glucose ≥ 200 mg/dl with symptoms such as polyuria, polydipsia, polyphagia
  - fasting blood glucose ≥ 126 mg/dl
2. Patients with periodontal disease having
  - generalized clinical attachment loss≥ 5mm measured with a Williams periodontal probe
  - bleeding on probing
3. Controls who are periodontally healthy
4. Patients who had not undergone any periodontal surgery for at least six months prior to sampling
5. All measurements and samples are taken before starting any periodontal therapy

#### Exclusion criteria

1. History of any antibiotic therapy within six months prior to study
2. History of any systemic disease for the control group
3. History of any systemic disease other than diabetes for the test group
4. Subjects who are pregnant and pre eclamptic
5. Subjects with a history of smoking, tobacco consumption
6. Subjects with vitamin supplements
7. Subjects who regularly use mouth washes

### Investigations

Venous blood samples were collected and centrifuged at 3000 rpm for 15 minutes and the supernatant serum is collected to measure the vitamin c, zinc and copper levels. The vitamin C levels of clinical samples are measured using spectrophotometric quantitation (dinitrophenly hydrazine method) and zinc and copper levels is measured using atomic absorption spectrophotometry

## RESULT

A study was conducted at the Department of Periodontics, A.B. Shetty Memorial Institute of Dental Sciences, Mangalore to evaluate and compare the serum micronutrient status (vitamin C, zinc and copper) in diabetic patients and healthy individuals with periodontitis.

**Graph 1:**
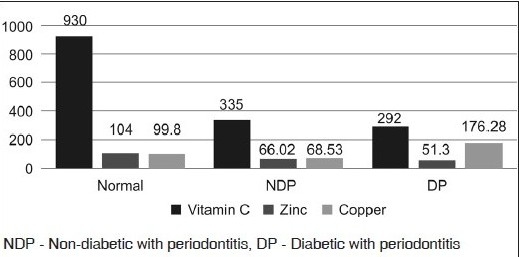
Micronutrient status in normal and periodontal complications with and without diabetes

The student *t* test was used for statistical analysis. Graph 1 shows the micronutrient status in normal and periodontally diseased individuals with and without diabetes.

The group statistics for vitamin C levels showed a mean of 0.292±0.004 for diabetic patients with periodontitis and a mean of 0.335±0.5 for healthy individuals with periodontitis. The statistical analysis showed a statistically significant decrease in vitamin C levels in diabetic patients with periodontitis when compared to healthy individuals with periodontitis with a *P* value less than 0.0001 [Table 1].

**Table 1:** Micronutrient status in normal and periodontally diseased individuals with and without diabetes

Parameters	Healthy individuals	Healthy with periodontitis	Diabetic with periodontitis	*P* value
Vitamin c	0.93±0.24	0.335±0.5	0.292±0.004	<0.001
Zinc	104.3±8.9	66.02±4.3	51.3±14.55	<0.001
Copper	99.8±19.1	68.53±7.03	176.78±6.67	<0.001

The statistical analysis for zinc levels showed a mean of 51.3±14.55 for diabetic patients with periodontitis and a mean of 66.02± 4.3 for healthy individuals with periodontitis. The results showed a statistically significant decrease in serum zinc levels in diabetic patients with periodontitis when compared to healthy individuals with periodontitis with a *p* value less than 0.0001 [Table 1].

The group statistics for copper levels showed a mean of 176.78±6.67 for diabetic patients with periodontitis and a mean 68.53±7.03 for healthy individuals with periodontitis. The statistical analysis showed a statistically significant increase in serum copper levels in diabetic patients with periodontitis when compared to healthy individuals with periodontitis with a p value less than 0.0001 [Table 1].

## DISCUSSION

The present study was conducted in the Department of Periodontics, A. B. Shetty Memorial Institute of Dental Sciences, Mangalore to evaluate and compare the micronutrient levels [vitamin C, zinc and copper] in diabetic patients and healthy individuals with periodontitis.

On a global basis, malnutrition is the most wide spread cause for immunosuppression in humans. Several lines of evidence strongly suggest that chronically malnourished individuals constitute a special risk group for severe and at times unique periodontal pathologies. The commonest types of periodontal lesions are inflammatory lesions elicited by specific pathogens in dental plaque. When exposed to infections or inflammatory agents, the host responds not only by mounting appropriate specific and nonspecific immune responses but also by initiating a well characterized series of metabolic adjustments. Inflammatory stimuli from dental plaque promote release of reactive free radicals and also elicit metabolic changes that are modulated by potent soluble mediators known as cytokines

Deficiency of vitamin C, zinc and copper increases susceptibility to infection, impair the function of neutrophils and macrophages, reduces antibody -mediated, cell- mediated, phagocytic and delayed type of hypersensitivity reactions and depletion of antioxidants.

The pathogenesis of periodontal disease is complex because it reflects a combination of the initiation and maintenance of the chronic inflammatory process by a diverse microbial flora and its numerous bacterial products. The subsequent host response to this infection mediates a complex cascade of tissue destructive pathways.

Additional factors contributing to this multifaceted local disease process in the oral cavity include a number of systemic diseases, especially diabetes that can exaggerate the host response to the local microbial factors, resulting in unusually destructive periodontal breakdown.

An abundance of information accumulated from studies on the complications of diabetes and periodontal disease has revealed that a hyperactive innate immune response may be the antecedent of both diseases, which probably have a synergistic effect when they coexist in the host.

Experimental evidence in diabetes mellitus patients has suggested that micronutrient deficiency leads to glucose intolerance. Serum content of copper increased and vitamin C and zinc levels were lower in diabetics than non-diabetics. These alterations may contribute to some of the complications of diabetes and there is a place for judicious replacement of micronutrients in diabetic patients with demonstrated deficiencies.

In our study the results show that serum vitamin C and zinc levels were lower and copper levels were higher in diabetic patients with periodontitis when compared to healthy individuals with periodontitis.

## CONCLUSION

It is reasonable to consume a nutritionally adequate diet to help maintain host resistance and the integrity of the periodontal tissues. A good diet contributes to both good general health and good oral health. There is, however, insufficient evidence to justify treatment with vitamin and mineral supplementation in the adequately nourished individual. Periodontal disease is an infectious disease that can be treated and prevented by the elimination of dental plaque in the adequately nourished individual. However, it may be reasonable to suggest vitamin and /or mineral supplement for patients whose nutrition might be inadequate. Future research should focus on an evaluation of which nutrients may help to prevent the onset and the progression of periodontal disease.
